# Comprehensive dataset for olive tree varieties responses to different level of drought stress

**DOI:** 10.1016/j.dib.2025.112026

**Published:** 2025-09-01

**Authors:** Kaloma Usman Majikumna, Mhamed Zineddine, Musa Mustapha, Liron Friedman, Eran Kaufman, Ahmed El Hilali Alaoui

**Affiliations:** a Euromed University of Fes, UEMF, Morocco; b Faculty of Engineering, Shenkar College of Art and Design, Israel

**Keywords:** Olive trees, Precision agriculture, Climate change, Drought stress, Irrigation, Growth response, Dataset

## Abstract

This study presents a detailed dataset on the growth responses of 80 young olive trees from three varieties: Haouzia and Menara (Moroccan) and Languedoc (French), under varying levels of water stress. The experiment was organised into eight groups: four experimental (A1, B1, C1, D1) and four control (A2, B2, C2, D2), with each group comprising 10 olive trees (4 Languedoc, 3 Menara, and 3 Haouzia). The olive trees were subjected to four distinct irrigation regimes, with groups A1 and A2 receiving 100 % of the water requirement, groups B1 and B2 receiving 50 %, groups C1 and C2 receiving 25 %, and groups D1 and D2 receiving no irrigation (0 %). Weekly data collection included trunk diameter, tree height, number of branches, SPAD values, leaf temperature, canopy cover, and individual tree images. This dataset offers valuable information for prospective researchers working on the impacts of drought stress on different varieties of olive trees, facilitating comparative analysis at varying stress levels. It is also useful for farmers, providing insights that can help optimize irrigation strategies and improve drought resilience in olive tree cultivation. The dataset can also be used to develop predictive artificial intelligence models for monitoring and analyzing drought stress in olive trees.

Specifications Table

**Table:** 

Subject	Agricultural Sciences (Agriculture Engineering)
Specific subject area	Agrotech, precision agriculture, smart agriculture, pattern recognition, climate change, drought response
Type of data	Raw olive trees phenology data stored as ‘.xlsx’ format and corresponding top and side view images stored as “.jpg” format.
Data collection	SPAD meter, measure tape and digital clipper tools was used for taking measurement of individual olive tree on weekly basis and recorded the data including and images on customized Kobo tool app on Android device, data recording was performed from March to July.
Data source location	Data acquisition was carried out in the olive field at the UEMF Experimental Precision Agricultural Farm, Euromed University of Fes, Morocco • City/Town/Region: Fes-Meknes, Fes • Country: Morocco • Latitude and longitude: 34.046917, −5.067606.
Data accessibility	Repository name: Mendeley Data Data identification number: DOI: 10.17632/22j26tpk63.1 Direct URL to data: [https://data.mendeley.com/datasets/22j26tpk63/1]
Related research article	None.

## 1 Value of the Data

• Provides a comprehensive dataset on how different olive tree varieties respond to varying drought stress levels, offering insights into the stress tolerance of young olive trees.
• Enables comparisons between two Moroccan olive varieties (Haouzia and Menara) and one French variety (Languedoc) under the same environmental conditions.
• Offers weekly records of essential images, growth parameters, including trunk diameter, height, number of branches, SPAD values, and leaf temperature, to analyze growth trends over time.
• Serves as a foundational dataset for future studies aimed at improving drought-resistant olive cultivars.
• Can be applied to develop artificial intelligence models that predict the impact of different irrigation treatments on young olive trees.
• Provides valuable insights for breeders and agronomists focused on selecting or developing olive varieties with better drought resistance.
• While chlorophyll fluorescence parameters are valuable for detailed physiological analysis, our use of SPAD measurements provides a rapid, non-destructive proxy for chlorophyll content, which, combined with visual image data, effectively captures drought stress responses relevant for practical applications.
• The image data captures visual signs of drought stress, such as leaf color changes, wilting, and canopy density, which complement numerical measurements, enabling more accurate and early detection through advanced multimodal AI analysis that benefits researchers, farmers, and decision-makers.
• Young olive plants are included because their early developmental stages are especially sensitive to drought stress, making them ideal for studying initial physiological and morphological responses. This focus supports early detection and management strategies to improve plant survival and productivity.

## 2 Background

The olive tree is a historically significant crop cultivated for centuries in the Mediterranean region, playing a vital role in the local economy and culture [1]. However, this region is also a hotspot for climate change, characterized by low precipitation and increasing temperatures [2,3]. As a result, olive tree cultivation is increasingly threatened by the impacts of climate change, particularly drought stress, with serious implications for Zero Hunger, Decent Work and Economic Growth, and Climate Action. According to Brito et al. [1], the forecasted future climatic conditions in the Mediterranean will negatively affect the physiology and yield of olive trees. Despite growing recognition of the need to address drought stress in olive trees, there is a significant shortage of field datasets on young olive trees subjected to different level of drought stress. The existing datasets, such as [4] focus on hyperspectral images of olive trees throughout the season, but are not specific to drought stress. Moreover, many existing studies such as [5] are based on simulations or greenhouse conditions in a highly controlled environment which may not accurately represent real-world scenarios. To obtain accurate and in vivo results, field experiments, not just simulated evaluations, are essential [6]. The creation of this dataset aims to fill that gap, providing a crucial resource for researchers investigating the drought stress of olive trees in the Mediterranean region. This new dataset will serve as a foundational reference for future studies, enabling more targeted and informed research in this critical field.

## 3 Data Description

**Fig. 1:**
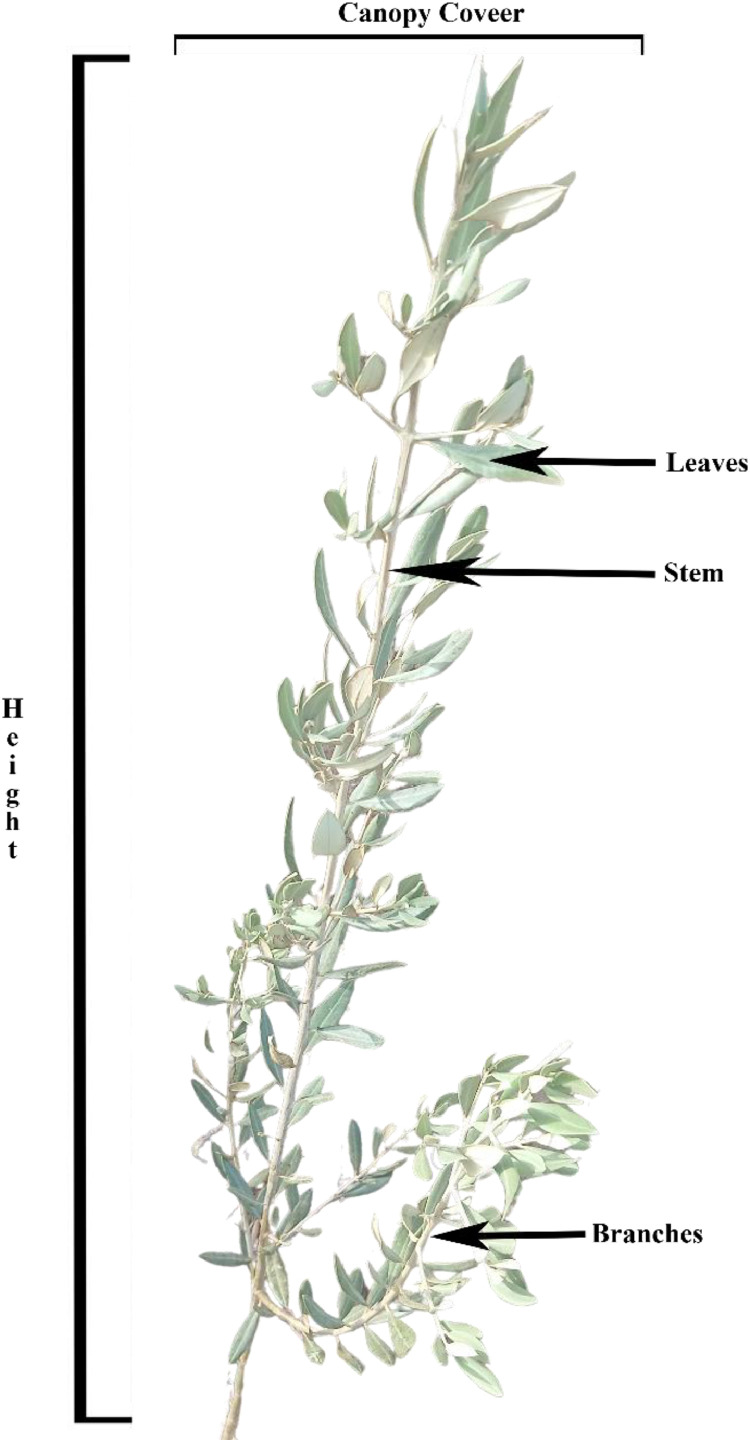
Olive tree.

This dataset includes physiological, growth, and image data from 80 young olive trees, organized into 4 irrigation groups replicated twice (8 groups in total) of 10 trees each, representing 3 different varieties[7]. Data collection occurred weekly for 19-weeks period from March to July 2024, with each session lasting 5 - 6 h. Each tree was assigned a unique ID for identification using the following format [group: variety: number]. The group names are A1, A2, B1, B2, C1, C2, D1, D2, and the varieties are Languedoc (L), Haouzia (H), and Menara (M). For example, "A1H3″ refers to group A1, Haouzia, and tree no 3. This ID system ensures data consistency and prevents misidentification. Fig. 1 shows the different parts of olive tree where the growth trait parameters measurement was taken.

The three olive varieties, Languedoc, Haouzia, and Menara, were selected due to their regional significance and contrasting drought tolerance, with Languedoc being a well-known French variety and Haouzia and Menara widely cultivated in Morocco. The experiment began in March when the young plants were in early vegetative growth stages and ended in July, just before the peak summer drought period, allowing assessment of physiological responses during critical growth and stress phases. This monitoring period was chosen to capture the transition from early growth to increasing drought pressure typical of the region. Before transplantation to the experimental field, all plants were grown from seeds under controlled nursery conditions to ensure uniform development.

**Fig. 2:**
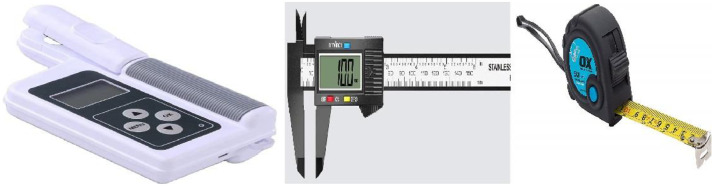
Data collection tools SPAD meter, digital clipper and measurement tape.

As shown in Fig. 2, the data collection tools utilized are: SPAD meter (model=TYS-B, year= 2023) to measure SPAD values and temperature, a tape measure for tree height, a digital clipper for trunk measurements, and the Kobo Collect app for data recording and image capture via Android phones. The data were periodically synced to the Kobo cloud.

The dataset is organized into two image folders and one Excel file. The Excel file, named “Growth.xls,” contains 1520 records with variables such as tree height, trunk diameter, canopy cover diameter, SPAD values (measured at three points), leaf temperature, branch count, irrigation regimes, and tree varieties as presented in Table 1. Irrigation regimes were calculated based on Penman-Monteith reference evapotranspiration, with Groups A1 and A2 receiving 100 % of the water requirement, Groups B1 and B2 receiving 50 %, Groups C1 and C2 receiving 25 %, and Groups D1 and D2 receiving 0 % (no irrigation).

**Table 1:** Data description.

S/N	Parameter	Data type	Unit	Data description
1.	Date	Date and time	Date	Indicates the data collection date
2.	Irrigation regime	categorical	%	Specifies the irrigation treatment level: 100 %, 50 %, 25 %, and 0 % of the irrigation demand.
3.	Group	Categorical	-	Indicates the experimental group the olive tree belongs, linked with a specific irrigation regime and other experimental conditions. Groups: A1, A2, B1, B2, C1, C2, D1, and D2.
4.	Variety	Categorical	-	olive tree varieties (e.g., Languedoc, Haouzia, Menara) use to assess response to different irrigation regimes.
5.	Olive ID	String	-	A unique identifier assigned to each olive tree in the experiment, ensuring accurate data tracking.
6.	Height	Numeric	-	Measured from the base to the top of the tree using a measuring tape
7.	Trunk Diameter	Numeric	mm	Diameter of the olive trunk, measured at breast height of 20 cm from ground using digital caliper
8.	Canopy cover	Numeric	Cm	Diameter of the olive tree’s canopy cover
9.	SPAD1	Numeric	-	Indicate the chlorophyll content in the leaves
10.	SPAD2	Numeric		Second measurement for accuracy
11.	SPAD3	Numeric	-	Third measurement for variability consideration
12.	Average_SPAD	Numeric	-	Average of the three SPAD value reading
13.	Temperature	Numeric	°C	Temperature of the leaves, indicator for the olive tree thermal response
14.	Branches	Numeric	Count	Number of branches of each olive tree, counted manually
15.	Top View image ID	Jpg	RGB	Top view images of the olive tree taken with an android phone via kobo collect.
16.	Side View image ID	Jpg	RGB	Side view images of the olive tree taken with an android phone via kobo collect.

**Fig. 3:**
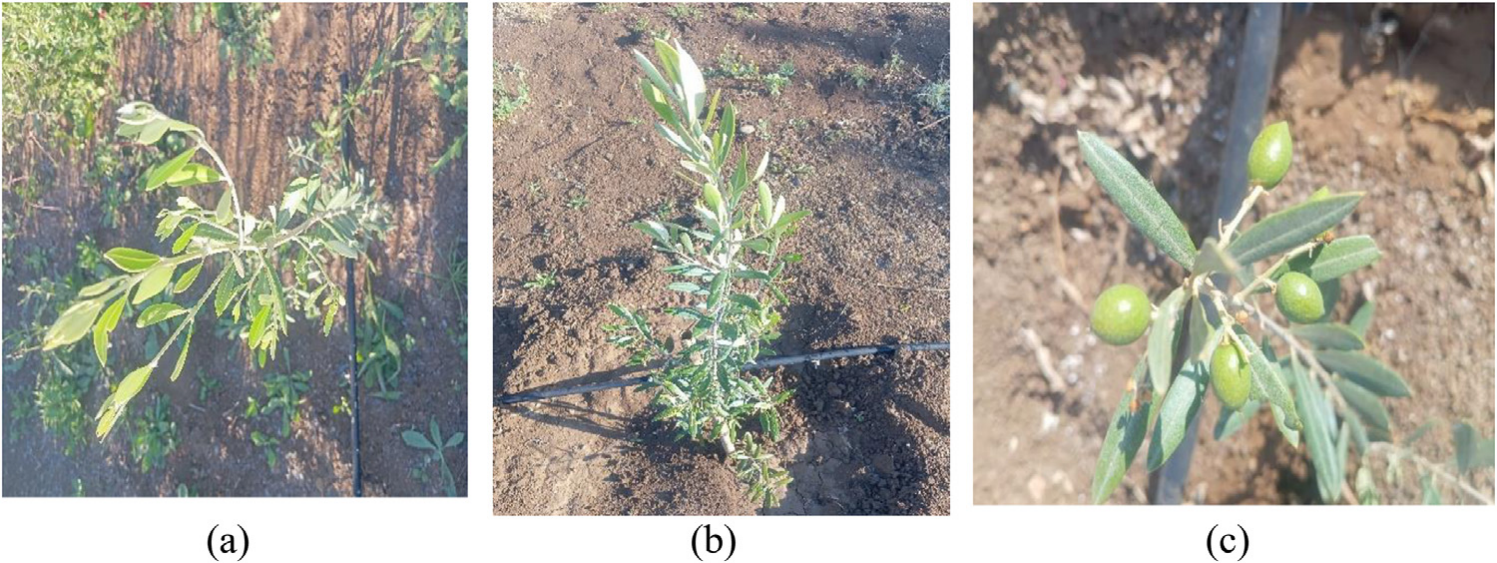
(a) top view, (b) lateral view, and (c) yield.

The Image data Is organized into two folders: “side view” (1440 RGB images of the olive trees’ side view), and “top view” (1440 RGB images of the trees’ top view). To ensure data accuracy, standardized protocols were followed, and measurements were validated by experienced agricultural researchers. Fig. 3 presents sample images from the dataset.

## 4 Experimental Design, Materials and Methods

**Fig. 4:**
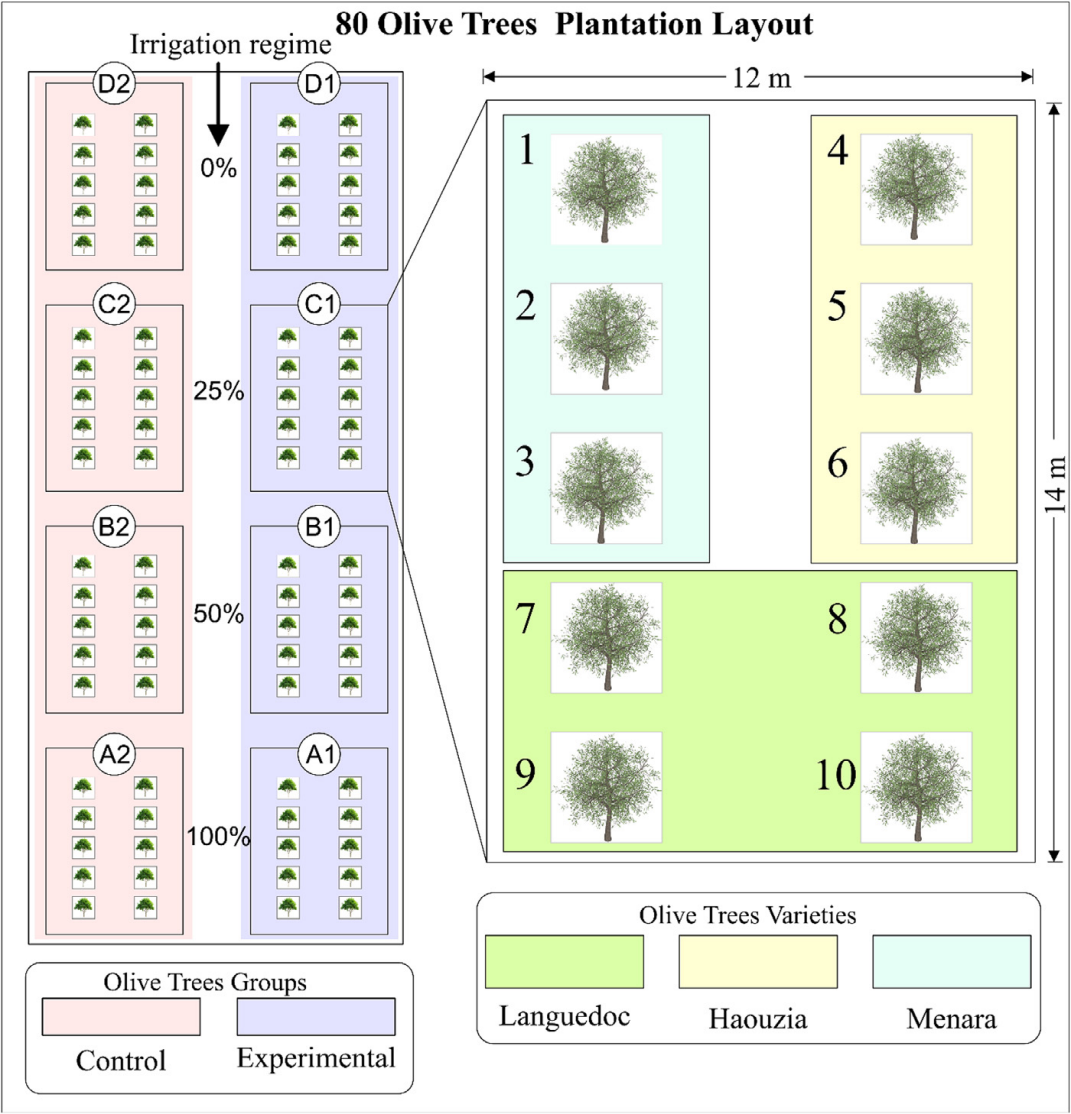
olive plantation layout.

The study employed a split-plot design to evaluate the impact of different drought stress levels on one-year-old olive trees. The layout of the 80-trees plantation, shown in Fig. 4, highlights the irrigation regimes and olive varieties with color coding. Each treatment group contained 10 trees from three varieties: Languedoc (4 trees per group), Haouzia (3 trees per group), and Menara (3 trees per group). These varieties were selected based on expert recommendations due to their prevalence in the region and their anticipated responses to drought stress. Each group was replicated, resulting in eight groups (A1, A2, B1, B2, C1, C2, D1, D2) to enhance reliability and allow for comparative analysis. The 80 olive tree plantations are arranged in groups, with a spacing of 3 m between each tree within a group, 6-meter gap between the control group and the experimental group, and a 10-meter gap between the cluster groups (A, B, C, D).

The 80 olive trees were divided into four distinct irrigation treatments:

- Groups A1 and A2: 100 % ET_C_
- Groups B1 and B2: 50 % ET_C_
- Groups C1 and C2: 25 % ET_C_
- Groups D1 and D2: 0 % ET_C_

ET_C_, representing olive tree water requirements, was calculated in equation (1).

(1) $$ET_{c}=ET_{o}*K_{c}$$

Where ET_O_ is derived from the Penman-Monteith equation [8], using climate data from the experimental weather station, as seeing in equation (2), and Kc is the crop coefficient of the olive tree.

(2) $$ET_{0}\;\begin{array}{c} =\;\frac{0.408*\Delta \left(Rs-G\right)+\gamma \frac{900}{Tmean+273}*u2*\left(es-ea\right)}{\Delta +\gamma \left(1+0.34u2\right)}\; \end{array}$$

Where:

**ET_0_** is the daily reference evapotranspiration (mm) day^−1^
**Rs** is the net radiation (MJ *m*^−2^) day^−1^
**Tmean** is the mean air temperature ( °C) at the height of 2m
**G** is the heat flux density (MJ *m*^−2^) day^−1^
**u2** is the windspeed(ms^−1^) at height of 2m
**es and ea** are the saturation and actual vapour pressure (kPa) derived from relative humidity
**∆** is the slope of the vapour pressure curve (kPa °*C*^−1^)
**γ** is the psychrometric constant (kPa °*C*^−1^).

Drip irrigation was installed, connected to an irrigation controller (Rain bird ESP-TM2, 2023), and a 2000 Liters buffer water tank, ensuring precise water delivery according to each group’s assigned irrigation regime. The system was carefully calibrated to maintain the designated ET_C_ levels, optimizing water use efficiency and minimizing wastage.

## Limitations

The dataset contains physiological and growth parameter data for the olive trees over 19 weeks (Excel file), but includes corresponding image data for only 18 weeks (raw images), as image data from the first week was not captured due to experimental constraints

## Ethics Statement

The authors have read and follow the ethical requirements for publication in Data in Brief and confirmed that the current work does not involve human subjects, animal experiments, or any data collected from social media platforms.

## CRediT authorship contribution statement

**Kaloma Usman Majikumna:** Conceptualization, Methodology, Validation, Formal analysis, Investigation, Data curation, Writing – original draft. **Mhamed Zineddine:** Conceptualization, Methodology, Supervision, Project administration. **Musa Mustapha:** Investigation, Data curation, Methodology, Software, Writing – original draft. **Liron Friedman:** Conceptualization, Methodology, Writing – review & editing. **Eran Kaufman:** Conceptualization, Methodology, Writing – review & editing. **Ahmed El Hilali Alaoui:** Project administration, Methodology, Validation, Formal analysis, Supervision.

## Data Availability

Mendeley DataOlive Tree Varieties Responses to Different level of Drought Stress (Original data)
